# Peptide KSL-W-Loaded Mucoadhesive Liquid Crystalline Vehicle as an Alternative Treatment for Multispecies Oral Biofilm

**DOI:** 10.3390/molecules21010037

**Published:** 2015-12-25

**Authors:** Jéssica Bernegossi, Giovana Maria Fioramonti Calixto, Paulo Ricardo da Silva Sanches, Carla Raquel Fontana, Eduardo Maffud Cilli, Saulo Santesso Garrido, Marlus Chorilli

**Affiliations:** 1 School of Pharmaceutical Sciences, Sao Paulo State University, UNESP, Rodovia Araraquara-Jaú Km 01, Araraquara, SP 14800-850, Brazil; jebernegossi@hotmail.com (J.B.); giovana.calixto@gmail.com (G.M.F.C.); 2 Chemistry Institute, Sao Paulo State University, UNESP, Campus Araraquara, Araraquara, SP 14800-900, Brazil; iqsanches@gmail.com (P.R.S.S.); eduardocilli@gmail.com (E.M.C.); saulosantesso@iq.unesp.br (S.S.G.)

**Keywords:** liquid crystalline systems, oral cavity, biofilm, KSL-W, antimicrobial peptide

## Abstract

Decapeptide KSL-W shows antibacterial activities and can be used in the oral cavity, however, it is easily degraded in aqueous solution and eliminated. Therefore, we aimed to develop liquid crystalline systems (F1 and F2) for KSL-W buccal administration to treat multispecies oral biofilms. The systems were prepared with oleic acid, polyoxypropylene (5) polyoxyethylene (20) cetyl alcohol (PPG-5-CETETH-20), and a 1% poloxamer 407 dispersion as the oil phase (OP), surfactant (S), and aqueous phase (AP), respectively. We characterized them using polarized light microscopy (PLM), small-angle X-ray scattering (SAXS), rheology, and *in vitro* bioadhesion, and performed *in vitro* biological analysis. PLM showed isotropy (F1) or anisotropy with lamellar mesophases (F2), confirmed by peak ratio quantification using SAXS. Rheological tests demonstrated that F1 exhibited Newtonian behavior but not F2, which showed a structured AP concentration-dependent system. Bioadhesion studies revealed an AP concentration-dependent increase in the system’s bioadhesiveness (F2 = 15.50 ± 1.00 mN·s) to bovine teeth blocks. Antimicrobial testing revealed 100% inhibition of multispecies oral biofilm growth after KSL-W administration, which was incorporated in the F2 aqueous phase at a concentration of 1 mg/mL. Our results suggest that this system could serve as a potential vehicle for buccal administration of antibiofilm peptides.

## 1. Introduction

The oral cavity is one of the most complex ecosystems in the human body and houses hundreds of species of microorganisms including yeasts, bacteria, protozoa, and virus [1]. It has the perfect combination of factors for the development of biofilms, which can accumulate and result in diseases such as caries, gingivitis, and periodontitis. Biofilms grow on dental surfaces by sequential and orderly settlement of several oral bacteria, which are organized functionally within an extracellular matrix of polysaccharides. This forms a complex structure with equally complex dynamics [2,3]. The bacteria that are fixed on the surface behave differently than planktonic bacteria owing to differential gene expression, which can result in a 1000-fold increase in the antimicrobial resistance of biofilms [4,5].

The accumulation and persistence of biofilms in the buccal cavity may result in aggravation and loss of periodontal insertions, which damages the supporting structures of the teeth. This is due to the effect of local accumulation of dental biofilms on the body’s immune response, which consequently causes loss of the dental element. Clinical manifestations of the toxic effects of biofilms can be broadly divided into two categories, gingivitis and periodontitis, depending on the degree of involvement of the supporting tissue [6,7].

The antimicrobial decapeptide NH_3_^+^-Lys-Lys-Val-Val-Phe-Trp-Val-Lys-Phe-Lys-CONH_2_ (KSL-W) studied, was consulted on the combinatorial Peptide Library Technology, and demonstrates a range of antibacterial activities [8]. *In vitro* studies have shown that it directly prevents the development of oral biofilms formed by human salivary bacteria, and inhibits the growth of oral bacterial pathogens associated with periodontitis and oral biofilm development [9,10,11]. However, its mechanism of action is not yet fully elucidated [12]. The low substantivity of peptides to infected tissues in solution, diluting effects of saliva, and tongue movements hinder the complete elimination of microorganisms. Therefore, strategies targeted at mitigating this problem by increasing their availability at the site of action have been developed [13].

Novel delivery systems including nanostructured systems of release present a promising solution to the problem of peptide administration in the oral cavity. These alternative carriers protect the active ingredient against degradation and make its release at a specific location at a controlled rate possible [14,15,16,17,18]. Reports in the literature indicate that the incorporation of peptides into drug release systems may be a useful strategy, independent of the route of administration [19,20,21,22]. 

**Figure 1:**
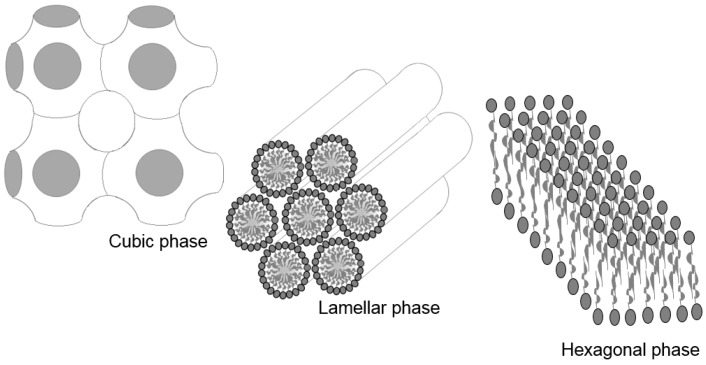
Liquid crystalline system (LCS) mesophases.

Among the nanostructured systems currently used for the incorporation of peptides, liquid crystalline systems (LCS) offer significant advantages, including controlled drug release, decreased thermal- or photo-degradation, and increased drug effectiveness [23]. The LCS structure has both liquid and solid properties, which include the rigidity and binding properties of solids as well as the mobility, cluttered areas, and fluidity of liquids. The main important and commonly observed lyotropic mesophases are the lamellar, hexagonal, and cubic ones, as shown in Figure 1 [24,25]. 

The structure of the cubic mesophase is the most difficult to visualize using polarized light microscopy, and it usually has a cubic symmetry with a dark field. The hexagonal phase consists of long aggregates with cylindrical arrangements that are detected under polarized light streaks while the lamellar mesophase consists of Maltese crosses, which are formed by surfactant bilayers separated by solvent layers [24,26,27,28]. Our research group extensively investigated PPG-5-CETETH-20, which belongs to the class of polyoxyethylene-polyoxypropylene surfactants. It is non-ionic and has been observed to form nanostructured systems of microemulsions and liquid crystals depending on the types and proportions of the oil phase (OP). Urban *et al.* [29] studied a combination of a surfactant and isopropyl myristate acetate used for incorporating dexamethasone acetate for topical application. The developed system exhibited high retention and permeation into the skin and therefore, is a promising vehicle for the delivery of topical corticosteroids. This surfactant has also been tested in systems containing water and oleic acid for the nasal administration of zidovudine. Low-viscosity systems (e.g., microemulsions) exhibit an interesting transition to liquid crystals when they come in contact with artificial nasal mucus, thereby increasing their bioadhesiveness to the mucosa. The system showed promising mucosal penetration, resulting in a more rapid absorption than observed with the oral administration of zidovudine syrup [30,31,32,33]. Another study with PPG-5-CETETH-20 involved the incorporation of microparticulate propolis into an LCS, which demonstrated prolonged release that would be advantageous for use in periodontal pockets [34,35]. Recent studies have demonstrated that different kinds of poloxamers including the P407, have advantageous properties such as increased drug adsorption into the epithelial cells [36]. Therefore, this study aimed to develop and characterize a buccal administration system of peptide KSL-W-loaded mucoadhesive liquid crystals as a treatment option for multispecies oral biofilms.

## 2. Results and Discussion

### 2.1. Peptide KSL-W Synthesis 

The chromatographic profile of purified peptide showed a single peak with a retention time of 9.2 min and relative purity of 96.4%. A theoretical molecular weight of 1308.7 g/mol was confirmed using electrospray ionization-mass spectrometry (ESIMS) with *m*/*z* = 436.6 [M + 3H]^3+^. 

### 2.2. Phase Behavior Studies

**Figure 2:**
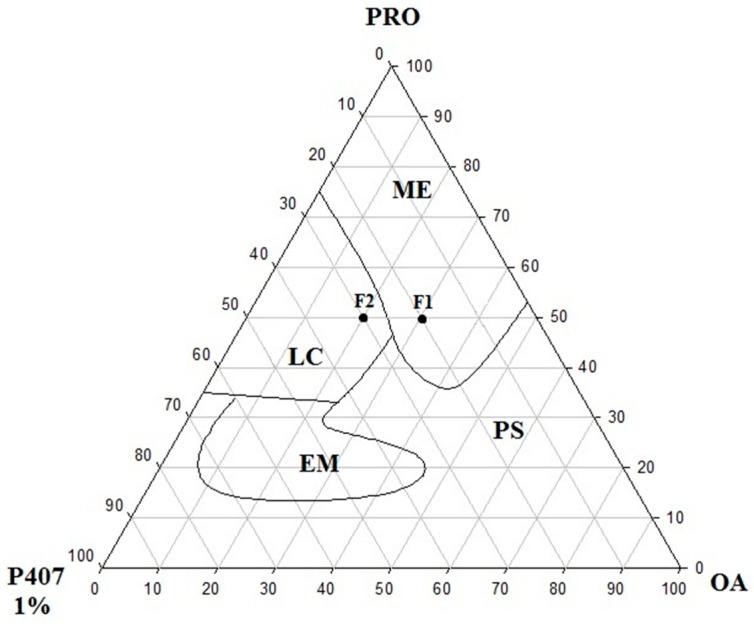
Ternary phase diagram of PPG-5-CETETH-20 (surfactant) with oleic acid (oil phase) and poloxamer 407 aqueous dispersion (1%). ME, microemulsion; LC, liquid crystal; EM, emulsion; PS, phase separation; PRO, PPG-5-CETETH-20 (Procetyl^®^); P407 1%, poloxamer 407 dispersion 1%; OA, oleic acid.

The diagram of the construct of the PPG-5-CETETH-20 surfactant (S), oleic acid oil phase (OP), and 1% poloxamer dispersion aqueous phase (AP) is depicted in Figure 2. The systems obtained were macroscopically classified as microemulsions, liquid crystals, emulsions, and phase separations. 

Microemulsion formulations were predominantly formed in the regions with lower proportions of the poloxamer 407 (AP) and PPG-5-CETETH-20 (S), 0%–25% and 35%–100% *w*/*w*, respectively) with 0%–48% (*w*/*w*) of the OP. Increasing the proportion of the AP (25%–65% *w*/*w*) allowed the development of liquid crystals in the formulations. While that decreasing the S and increasing the AP (15%–35% and 65%–85% *w*/*w*, respectively) produced the emulsion systems. Furthermore, there was no phase separation in a small region of the diagram.

### 2.3. Polarized Light Microscopy (PLM) and Small-Angle X-ray Scattering (SAXS)

**Figure 3:**
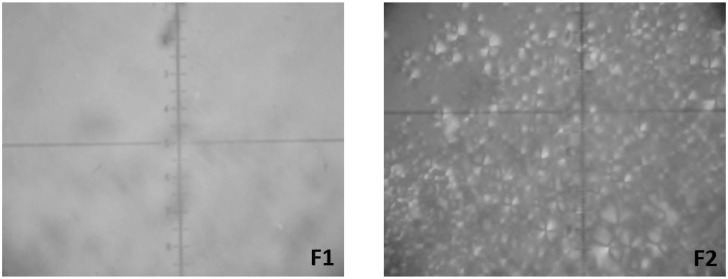
Photomicrographs obtained using polarized light microscopy (PLM) showing isotropy of microemulsion (F1) and Maltese cross of lamellar phase (F2).

The F1 and F2 formulations were selected for characterization as already pointed out in the phase diagram in Figure 1, and their compositions are listed in Table 1. The S concentration was fixed at 50% while the proportions of the AP and OP were varied. The polarized light microscopy (PLM) images are shown in Figure 3. 

**Figure 4:**
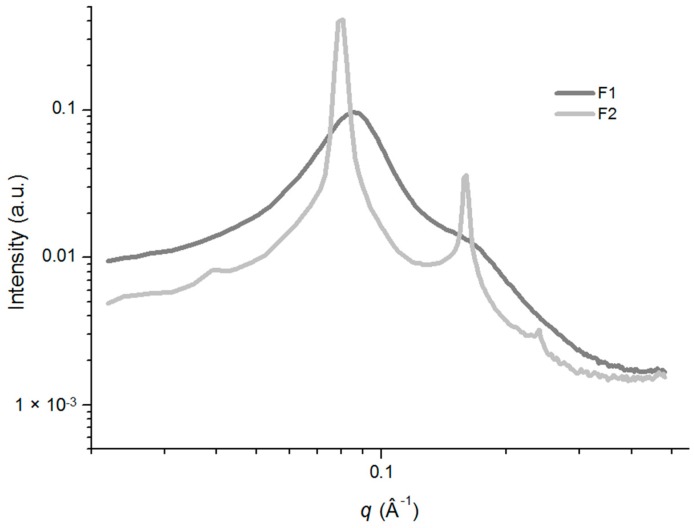
Small-angle X-ray scattering (SAXS) patterns of samples F1 and F2 without KSL-W. Data were collected at 25 °C.

PLM analysis of the F1 sample showed a dark field, and it was characterized as isotropic. Furthermore, its low viscosity suggested that it was a microemulsion system. In contrast, F2 was characterized as anisotropic, with evidence of Maltese crosses and lamellar mesophases that were present as lipid bilayers separated by one layer of water. The phase behavior of the selected systems was confirmed by using small-angle X-ray scattering (SAXS) analysis, and the scattering profile data were plotted as intensity (I) *vs.* the scattering vector (q).The resulting analysis of the SAXS curves are shown in Figure 4 and the relationship between the distances of the Bragg peaks on the q, which confirmed the structures observed in the PLM, are shown in Table 1.

**Table 1:** Calculated d values for formulations. Peak positions of scattering power of small-angle X-ray scattering (SAXS) curves and their respective correlation distances (d) and assignments for phase classification (d1/d2 and d1/d3).

Sample	q1 (Å^−1^)	q2 (Å^−1^)	q3 (Å^−1^)	d2/d1 (Å)	d3/d1 (Å)	nm
F1	0.08	-	-	-	-	-
F2	0.08	0.16	0.24	2	3	7.85

The application of this technique enabled the identification of the type of aggregation exhibited by the compounds by correlating the diffraction spikes on the shaft with the scattering vector (q). The micellar structure of the F1 formulation had a scattering profile that showed that the maximum intensity value of q was different from zero and exhibited a long tail. The F1 formulation is illustrated in Figure 4 [28].

The liquid-crystalline structure phases mandatorily adhere to a relationship consisting of a 1:2:3 ratio [37]. Formulation F2 (Figure 4) obeyed this ratio and therefore, had a lamellar mesophase structure. Increasing the AP in the systems caused the F1 formulation to transition from a microemulsion system to a lamellar mesophase (similar to F2), as observed using the PLM analysis. The liquid crystal system (F2) can be considered to have a nanostructured organization because the space between two adjacent layers was 7.85 nm and in the nanoscale range [28].

### 2.4. Rheological Analysis

**Figure 5:**
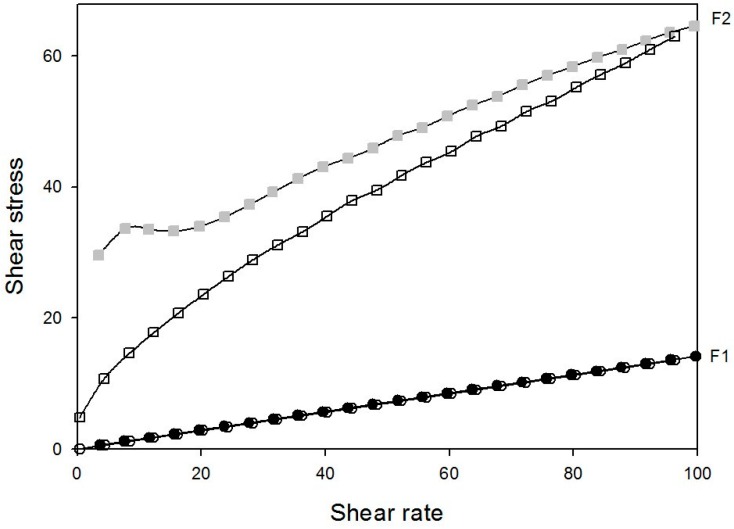
Rheograms of systems composed of poloxamer 407 dispersion (1%), oleic acid, and PPG-5-CETETH-20. Closed and open symbols indicate going and return, respectively. Data were collected at 37 °C.

Rheology testing of the streaming of substances can elucidate how a sample behaves when a specific voltage is applied [38]. The continuous shearing of samples F1 and F2 is displayed in Figure 5, while the ɳ and K values are shown in Table 2.

**Table 2:** Rheological parameters of hydrogels composed of poloxamer 407 dispersion (1%), oleic acid, and PPG-5-CETETH-20 systems.

Sample	N	K	G’	G’’
F1	0.986	0.150	0.29	2.49
F2	0.616	3.690	342.56	109.12

Sample F1 showed the characteristics of a Newtonian fluid with *n* values of approximately 1. However, F2 demonstrated non-Newtonian fluid characteristics and was pseudoplastic with thixotropic-type descending curves. The viscosity of the system increased with increasing proportion of the AP.

**Figure 6:**
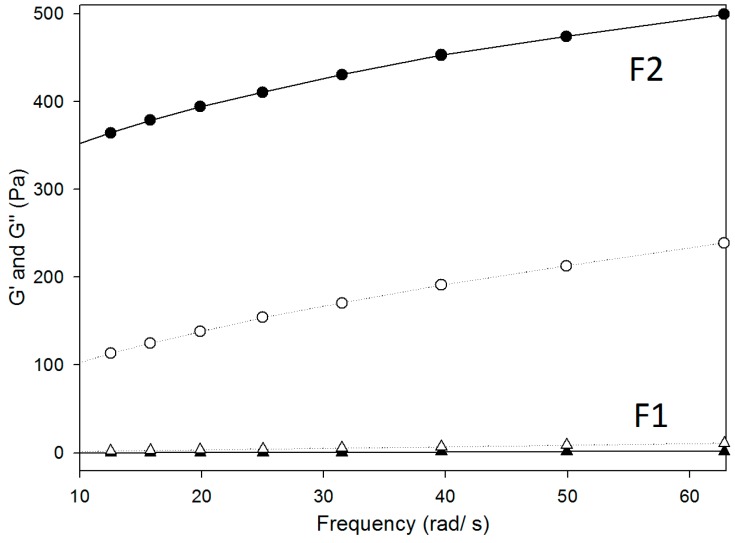
Frequency sweep profile of storage and loss moduli G’ and G” (closed and opened and symbols), respectively, of systems composed of poloxamer 407 dispersion (1%), oleic acid, and PPG-5-CETETH-20. Data were collected at 37 ± 0.5 °C.

Pseudoplastic behavior is desirable for pharmaceutical applications because the flow property after application of a stress force is excellent, which facilitates the administration of the formulation using a syringe, which is a convenient method for the patient [39]. The oscillatory rheological data are displayed as storage and loss moduli (G’ and G’’, respectively), as a function of the oscillatory frequency in Figure 6 and Table 2. The G’ is the elastic module, which represents both energy stored during deformation when the voltage increases and energy released when the tension is lost. In contrast, the G’’ is the viscous module (element) that cannot store energy [40,41].

The frequency sweep analyses demonstrated that F1 was more viscous than it was elastic because the G’’ was higher than the G’ was, indicating that it was more organized. In contrast, sample F2 demonstrated more elasticity than viscosity. These factors are related to bioavailability, and compared to viscous formulations; those with elasticity remain longer in the buccal mucosa membrane, which increases the drug absorption time [42].

### 2.5. Bioadhesion Studies

**Figure 7:**
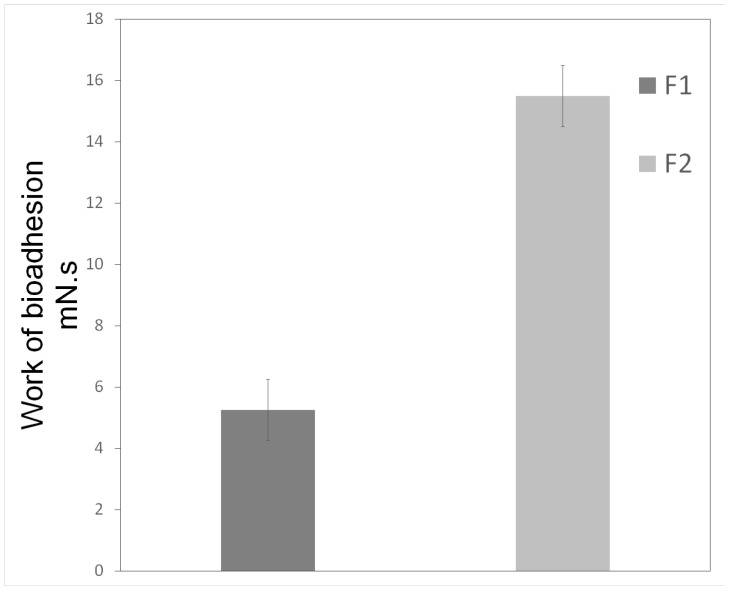
Parameters of *in vitro* bioadhesion test of systems composed of poloxamer 407 dispersion (1%), oleic acid, and PPG-5-CETETH-20. Data were collected at 37 ± 1°C and values represent mean ± standard deviation (SD) of seven replicates.

The results of the bioadhesion study are shown in Figure 7. The F1 formulation required less force to penetrate break the bond between the block of bovine teeth and the formulation compared with the F2 formulation (5.25 ± 1.00 and 15.50 ± 1.04 mN·s, respectively). Furthermore, increasing the proportion of the AP produced an F2 formulation that was more viscous and bioadhesive than F1 was.

However, the F1 formulation presents advantages for oral application over the F2 since its fluidity favors clinical application. In addition, F1 also becomes more viscous than F2 following contact with saliva. This incorporation of saliva renders the F1 load structural similar to that of the F2 formulation. The F1 formulation exhibited an increased retention time and, therefore, would likely be resistant to possible washout by salivary flow as well as mechanical interferences caused by chewing and speaking.

These results show that the greater the proportion of water, the more viscous the system became. Furthermore, this also increases the mucoadhesive characteristics, which is more advantageous pharmaceutically since the system would have longer contact at the desired location, thereby increasing drug absorption and ultimately enhancing the clinical performance of the formulation [42].

### 2.6. Determination of Activity against Multispecies Oral Biofilm 

The results obtained in the biofilm inhibition test are shown in Table 3. The colony forming unit (CFU) count of the formulation-treated and the positive control groups were compared (251 CFU was considered 100%). 

**Table 3:** Average colony forming unit (CFU %) ± standard deviation (SD) for KSL-W peptide-loaded F2 liquid crystalline system (LCS) against multispecies oral biofilm (*n* = 2). P, KLS-W in solution at 1 mg/ mL; F2-P, F2 formulation with 1 mg/mL of peptide KSL-W; F2, F2 without peptide KSL-W.

Samples	Growth CFU (%)
Positive control	100 (5.1)
Negative control	0 (0)
P	9.98 (1.2)
F2-P	0 (0)
F2	14.37 (1.8)

Chlorhexidine digluconate is frequently used in oral solutions as an active ingredient against pathogenic microorganisms in the oral cavity and therefore, was used as the negative control. In this study, we tested the antimicrobial activity of an LCS containing KSL-W against multispecies oral biofilms, which are considered one of the leading causes of human buccal diseases. The F2-P formulation (0% growth) presented promising results compared with the positive control. Compared with the P (9.98% growth) with the same peptide concentration, the formulation exhibited greater effectiveness, indicating possible synergism between the formulation and the incorporated peptide. To elucidate whether the observed controlled release contributed to the efficacy of the formulation, a peptide-release test was built into the system and evaluated. 

Leung *et al.* [43] incorporated KSL-W peptide into chewing gum with an additional component, cetylpyridinium chloride (CPC), and observed its action on salivary biofilms grown on hydroxyapatite discs. The results led them to conclude that there was a synergistic interaction between the components of the chewing gum and the CPC antimicrobial peptide. Furthermore, they found that it is possible to decrease the concentrations of both antimicrobial components and obtain the same effect. In contrast, the positive controls showed that over 90% of the biofilm was eliminated after treatment with the test formulation containing the lowest amount of the active substance (200 and 25 µg/mL of peptide and CPC, respectively) [43]. Analysis of F2 without the antimicrobial KSL-W peptide (14.37% growth) showed a considerable reduction in biofilm formation, which may have been caused by the action of the S on the membrane of the microorganisms present in the biofilm [44].

## 3. Experimental Section

### 3.1. Materials

The PPG-5-CETETH-20 was kindly provided by Croda^©^ Procetyl AWS™ (Campinas, Sao Paulo, Brazil). The oleic acid acquired as Synth^®^ (Diadema, Sao Paulo, Brazil) and poloxamer 407 were from Sigma-Aldrich (St. Louis, MO, USA) High-purity water was prepared using a Millipore Milli-Q plus purification system. Chlorhexidine gluconate was obtained from Periotrat^®^ (Porto Alegre, Brazil) Menadione, hemin, medium tryptone, soy agar, and tryptone soy broth were purchased from Sigma-Aldrich. The KSL-W peptide was synthesized at the Institute of Chemistry, UNESP (Universidade Estadual Paulista “Júlio de Mesquita Filho”) Araraquara, Brazil.

### 3.2. Peptide Synthesis

The synthesis of the peptide sequence NH_3_^+^-Lys-Lys-Val-Val-Phe-Trp-Val-Lys-Phe-Lys-CONH_2_ (KSL-W) was performed using the solid-phase peptide synthesis (SPPS) method. The standard Fmoc (9-fluorenylmethyloxycarbonyl) protocol was employed using a Rink-MBHA resin [45] with a degree of substitution of 0.6 mmol/g. The experimental protocol was the same as previously reported by Crusca *et al.* [46]. Cleavage of the resin and removal of the side chain-protecting groups were performed simultaneously using trifluoroacetic acid (TFA), triisopropylsilane, and water (95:2.5:2.5, *v*/*v*/*v*, respectively). The crude peptide was precipitated with anhydrous ethyl ether, separated from the soluble nonpeptide material by centrifugation, extracted with 0.045% TFA in water (solvent A), and lyophilized. After dissolution in solvent A, the peptide purification was carried out using semi-preparative high-performance liquid chromatography (HPLC) using a reverse phase C18 column (25 × 2.12 cm). The flow rate was 5 mL/min and the ultraviolet (UV) detection was carried out at 220 nm. After purification, the peptide homogeneity was evaluated using analytical HPLC using a reverse phase column (25 × 0.46 cm, 300 Å pore size) and a linear gradient of 5%–95% (*v*/*v*) of 0.035% TFA/acetonitrile (solvent B) for 30 min, at a flow rate of 1.0 mL/min with UV detection at 220 nm. The identity of the peptide was confirmed using ESIMS and the final purity of the peptide was higher than 95%.

### 3.3. Ternary Phase Diagram Construction

To obtain the ternary phase diagrams, the S, OP and AP were combined at different weight ratios at room temperature (25 ± 2 °C). The percentages of the three components ranged from 10%–80% (*w*/*w*), and were calculated to obtain the points that defined the delimitations between the regions of the ternary phase diagram. After 24 h, the formulations were visually classified using phase separation, opacity or viscosity of the liquid systems, and translucency [24]. Two formulations were selected for further physicochemical characterization in the present study and are listed in Table 4.

**Table 4:** Formulation compositions.

	Composition % (*w*/*w*)	
Components	F1	F2
Oleic Acid	30	20
PPG-5-CETETH-20	50	50
Dispersion Poloxamer 407 1%	20	30

### 3.4. PLM

The samples for the PLM analysis were prepared by placing a drop of each formulation between a coverslip and a glass slide, followed by examination under polarized light at room temperature (25 ± 1 °C). An optical microscope (Axioskop^©^, Zeiss, Gottingen, Germany) was used to analyze the various fields of each sample. The isotropic or anisotropic behavior of the samples was observed, and images were acquired at 40× magnification at room temperature (25 ± 1 °C).

### 3.5. SAXS

Data were collected using the Synchrotron SAXS beamline at the National Laboratory of Synchrotron Light (LNLS, Campinas, Brazil) in an SAXS1 station. The line was equipped with a monochromator (λ = 1.488 Å), vertical detector located approximately 1.5 m from the sample, and multichannel analyzer, which registered the *I* of the spreading q. The scattering of the mica and air were subtracted from the total scattered *I*. The recording of each spectrum was performed in approximately 45 s, and this equipment allowed the calculation of the scattering vector, q, at approximately 0.1–2.3 Å^−1^. The test was performed with the formulations at 37 ± 1 °C [28,47,48].

### 3.6. Rheological Analysis

The rheological analysis was performed at 37 ± 0.5 °C using a controlled stress rheometer (AR 2000EX, TA Instruments, New Castle, DE, USA) with parallel plate-plate and cone-plate geometry, according to the consistency of each formulation. The samples were placed carefully on the lower base and allowed to equilibrate for at least 5 min prior to the analysis. The continuous analysis was performed using a shear rate of 0–100 s^−1^ for the upslope and 100–0 s^−1^ for the downward curve over 120 s. The oscillatory formulation analysis was performed after determination of the linear viscoelastic region where the stress is directly proportional to strain and the storage modulus is constant. The frequency sweep analysis was performed over a frequency range of 0.1–10 Hz after the application of a constant stress of 1 Pa stress. The continuous and oscillatory analyses were carried out in triplicate. The flow and consistency indices were estimated from the Power law described in Equation (1) [16,49] for a quantitative analysis of flow behavior [28,50,51,52]:

τ = k × γ^n^ (1)

where, “τ” is the shear stress, “γ” is the shear rate, “k” is the consistency index, and “n” is the flow index.

### 3.7. Bioadhesion Studies

The bioadhesive force between the bovine tooth block moistened with saliva and the LCS was assessed using a TA-XTplus texture analyzer (Stable Micro Systems, Godalming, UK) in adhesion test mode. The bovine teeth were obtained under supervision of the Brazilian Ministry of Agriculture, and stored in a refrigerator in distilled water. The blocks were prepared using a high-speed motor (Dabi Atlante^®^, Ribeirão Preto, Brazil) coupled to a diamond disk (KG Sourensen^®^, Cotia, Brazil). A block with a diameter and thickness of 1 and 0.30 cm, respectively, was used as the cement region while saliva was obtained from 20 volunteer donors and sterilized using a 0.22-μm porous membrane [53]. The collection of the saliva samples was authorized by the Research Ethics Committee of the School of Pharmaceutical Sciences, UNESP, Araraquara, SP, Brazil (Process 423.890/2013), and the volunteers provided their informed consent. The bovine tooth block was fixed to a cylindrical probe (10 mm diameter) with adhesive tape (Adermax^®^, Sumaré, Brazil), and moistened with saliva. The probe was lowered at a constant speed (1 mm/s) until the tooth block and sample made contact for 60 s, and then was displaced, rising at a velocity of 0.5 mm/s until there was contact between the surfaces [54]. This process was replicated seven times at 37 ± 1 °C.

### 3.8. Determination of Activity against Multispecies Oral Biofilm 

The multispecies oral biofilm was grown according to the method of Fontana *et al.* [55] in an enriched agar medium containing 20 g/L trypticase soy agar (Oxoid Ltd., Basingstoke, Hampshire, UK), 26 g/L brain-heart infusion agar (Difco Laboratories, Detroit, MI, USA), 10 g/L yeast extract (BBL), and 5 mg/L hemin (Sigma-Aldrich Chemical Corp.). The medium was autoclaved at 121 °C for 20 min and then cooled to 50°C. Menadione (5 mg/mL, Sigma-Aldrich Chemical Corp.) was added under aseptic conditions. Aliquots of the hot agar mixture were dispensed into 96-well microtiter plates Techno Plastic Products, TPP AG, Trasadingen, Switzerland) at 150 µL/well and allowed to cool and dry. The saliva, which was used for the formation of the multispecies oral biofilm, was collected from volunteers who were not allowed to perform any oral hygiene practices (brushing or flossing) and were asked to fast during the 12 h prior to saliva sampling. The entire saliva sample was dispersed under aerobic conditions and added to the trypticase soy broth (TSB, Beckton, Dickinson and Company, Sparks, MD, USA) at a ratio of 1:1 (saliva:broth). For biofilm development, the saliva/TSB inoculum contained approximately 10^7^ cells/mL, and 150 µL of this inoculum (approximately 1.5 × 10^6^ bacteria) was carefully pipetted and used to fill the enriched agar wells in each 96-well plate. The plates were then incubated aerobically at 35 ± 1 °C for 7 days. After an initial incubation period of 48 h, the liquid medium was carefully aspirated from each well and the biofilms were replenished with fresh TSB, which was slowly added daily to each well, to avoid disrupting the biofilm [55]. The peptide treatment was applied to the biofilm model to determine its effect. Five groups were tested: TSB enriched with menadione and hemin (positive control), chlorhexidine gluconate (1.2 mg/mL, negative control), KSL-W aqueous solution (1 mg/mL), F2 loaded KSL-W (1 mg/mL), and F2 without KSL-W. 

On day 4 of biofilm growth, the test samples were incubated for 24 h. On day 7 of their development, the biofilms were gently scraped from the enriched agar in each well using a sterile bacteriological loop to remove the entire visible biomass. After treatment, the bacterial suspensions were serially diluted in TSB, 100-µL aliquots were plated on the enriched agar plates, and then they were incubated under aerobic conditions for 7 days. Survival fractions were evaluated by quantifying the number CFU using repeated measures analysis of variance (ANOVA) to compare treatment groups. The values were expressed as a percentage of the total number of colonies grown in the positive control, which was considered 100%.

## 4. Conclusions

The mixture of PPG-5-CETETH-20, oleic acid, and poloxamer 407 dispersion (1%) exhibited the capability to form microemulsions and liquid crystalline lamellar phase systems. Furthermore, we observed that the addition of larger proportions of water contributed to creating an LCS with a more organized structure starting a microemulsion. In addition, the LCS formed was a suitable platform for the incorporation of the KSL-W peptide (F2-P) and had good antimicrobial activity compared with the aqueous peptide solution, and its effect was comparable to that of chlorhexidine digluconate (negative control). Therefore, this system could serve as a potentially effective alternative treatment for multispecies oral biofilms.
